# Molecular features of untreated breast cancer and initial metastatic event inform clinical decision-making and predict outcome: long-term results of ESOPE, a single-arm prospective multicenter study

**DOI:** 10.1186/s13073-021-00862-6

**Published:** 2021-03-15

**Authors:** Céline Callens, Keltouma Driouch, Anaïs Boulai, Zakia Tariq, Aurélie Comte, Frédérique Berger, Lisa Belin, Ivan Bièche, Vincent Servois, Patricia Legoix, Virginie Bernard, Sylvain Baulande, Walid Chemlali, François-Clément Bidard, Virginie Fourchotte, Anne Vincent- Salomon, Etienne Brain, Rosette Lidereau, Thomas Bachelot, Mahasti Saghatchian, Mario Campone, Sylvie Giacchetti, Brigitte Sigal Zafrani, Paul Cottu

**Affiliations:** 1 Genetics Department, Institut Curie, PSL Research University, Paris, France; 2 Department of Medical Oncology, Institut Curie, PSL Research University, 26 rue d’Ulm, 75005 Paris, France; 3 Department of Biostatistics, Institut Curie, Saint-Cloud, France; 4 Imaging Department, Institut Curie, PSL Research University, Paris, France; 5 Institut Curie Genomics of Excellence (ICGex) Platform, Institut Curie Research Center, PSL Research University, Paris, France; 6 Surgery Department, Institut Curie, Paris, France; 7 Pathology and Tumor Biology Department, Institut Curie, PSL Research University, Paris, France; 8 Medical Oncology, Institut Curie, Saint-Cloud, France; 9 Centre Léon Bérard, Lyon, France; 10 Gustave Roussy Cancer Campus, Villejuif, France; 11 Institut de Cancérologie de l’Ouest Nantes, Nantes, France; 12 Hôpital Saint Louis, Breast diseases center, Paris, France

**Keywords:** Breast cancer, Metastasis, Next generation sequencing, Targetable genes, Prognosis, de novo metastases

## Abstract

**Background:**

Prognosis evaluation of advanced breast cancer and therapeutic strategy are mostly based on clinical features of advanced disease and molecular profiling of the primary tumor. Very few studies have evaluated the impact of metastatic subtyping during the initial metastatic event in a prospective study. The genomic landscape of metastatic breast cancer has mostly been described in very advanced, pretreated disease, limiting the findings transferability to clinical use.

**Methods:**

We developed a multicenter, single-arm, prospective clinical trial in order to address these issues. Between November 2010 and September 2013, 123 eligible patients were included. Patients at the first, untreated metastatic event were eligible. All matched primary tumors and metastatic samples were centrally reviewed for pathological typing. Targeted and whole-exome sequencing was applied to matched pairs of frozen tissue. A multivariate overall survival analysis was performed (median follow-up 64 months).

**Results:**

Per central review in 84 patients (out of 130), we show that luminal A breast tumors are more prone to subtype switching. By combining targeted sequencing of a 91 gene panel (*n* = 67) and whole-exome sequencing (*n* = 30), a slight excess of mutations is observed in the metastases. Luminal A breast cancer has the most heterogeneous mutational profile and the highest number of mutational signatures, when comparing primary tumor and the matched metastatic tissue. Tumors with a subtype change have more mutations that are private. The metastasis-specific mutation load is significantly higher in late than in de novo metastases. The most frequently mutated genes were *TP53* and *PIK3CA*. The most frequent metastasis-specific druggable genes were *PIK3CA*, *PTEN*, *KDR*, *ALK*, *CDKN2A*, *NOTCH4*, *POLE*, *SETD2*, *SF3B1*, and *TSC2*. Long-term outcome is driven by a combination of tumor load and metastasis biology.

**Conclusions:**

Profiling of the first, untreated, metastatic event of breast cancer reveals a profound heterogeneity mostly in luminal A tumors and in late metastases. Based on this profiling, we can derive information relevant to prognosis and therapeutic intervention, which support current guidelines recommending a biopsy at the first metastatic relapse.

**Trial registration:**

The trial was registered at ClinicalTrials.gov (NCT01956552).

**Supplementary Information:**

The online version contains supplementary material available at 10.1186/s13073-021-00862-6.

## Background

The therapeutic strategy in early breast cancer (BC) is based on TNM staging and on primary tumor (PT) molecular subtype as defined by expression of hormone receptors (estrogen, ER, and progesterone, PR), HER2, and to a lesser extent by Ki67 expression level and genomic signatures [1]. In the metastatic setting, the therapeutic strategy is mostly based on clinical data, such as delay of relapse, metastatic sites, age, and general condition [2]. A detailed subtype and genomic characterization of BC metastases could help at the individual patient level to specify prognosis and guide the choice of therapy, and several guidelines have implemented a recommendation for biopsy of BC metastases in order to confirm diagnosis and reassess tumor subtype [1, 3, 4]. A recent pooled analysis of 39 studies evaluating receptor conversion in BC metastases showed that ER and PR conversion (both ways) occurred in about 20% and 35% of patients, respectively, and HER2 conversion in about 10% of patients [5]. Of note, very few prospective studies, and only four with centralized pathology review have been reported [6–9]. The lack of prospective studies with sufficient post treatment follow-up, assessing the clinical consequences of receptor conversion, has also been underlined [5]. Moreover, recent metastatic sequencing studies have also begun to unravel the genomic complexity of BC metastases [10, 11] and to correlate the genomic alterations with outcome [10]. From a theranostic perspective, it has been shown that metastases may acquire driver genomic alterations involving key pathways such as *SWI-SNIF* or *JAK-STAT3* pathways [12], *MAPK* pathway in endocrine-resistant metastases [13], receptor tyrosine kinases activation [14], and specific oncogenes such as *ESR1* or *CDH1* [15]. It has nonetheless recently been strongly suggested that this genomic complexity, as captured in heavily treated metastatic relapses, could reflect the effect of treatments on the genome, rather than actually document the driving processes of the metastatic spread [11].

No large prospective study based on a homogeneous population of patients at first systemic relapse, including a systematic matched analysis of primary tumor (PT) tissue along with metastatic (M) tissue has been reported so far, adding heterogeneity to the results of previous studies and ultimately questioning their clinical utility. Within this context, we developed and implemented ESOPE (*c*hanges in phenotyp*e* and genotype of breast cancer*s* during the metastatic process and *op*timization of therapeutic targ*e*ting), a prospective, single-arm multicenter study designed to document prospectively phenotypic and genomic discordance between PT and M in a large population of patients at first metastatic relapse of breast cancer, before any systemic treatment for advanced disease. We describe here the molecular landscape of metastatic breast cancer at the very first, untreated, metastatic event and assess the long-term prognostic value of conventional biomarkers and genomic features of the metastatic tissue.

## Methods

### Patients and samples

This prospective study was conducted in 6 French academic centers (Institut Curie, Paris and Saint-Cloud—CHU Saint-Louis, Paris—Gustave Roussy Cancer Campus, Villejuif—Centre Léon Bérard, Lyon—Institut de Cancérologie de l’Ouest, Saint-Herblain—Centre Oscar Lambret, Lille) on patients with first BC metastatic event (either de novo or first relapse), before any treatment for metastatic disease, between November 2010 and September 2013. Inclusion criteria were as follows: women ≥ 18 years; PS ≤ 2; evaluable metastatic disease with a lesion accessible to biopsy, as evaluated by a radiologist or a surgeon in a multidisciplinary meeting, outside any previous radiation therapy field; social and psychological welfare in concordance with compliance to the study. At least one available FFPE PT sample was mandatory. Non-inclusion criteria were as follows: bilateral breast cancer; unilateral, multifocal breast cancer, with different pathological subtypes; isolated local or contralateral relapse. Solitary bone and/or brain metastatic disease unless metastatic sites were eligible for a therapeutic surgery and/or the only metastatic sites sampled for diagnosis purpose; previous history of gynecological cancer; any other cancer history in the 5 previous years, except curatively treated carcinoma of cervix, basal cell carcinoma, and squamous cell carcinoma of the skin; any coagulopathy contraindicating tumor biopsy, and presence of a contraindication to general anesthesia, if required. This study was approved by the French Ethics Committee (“Comité de Protection des Personnes”) and the Institutional Reviews Boards of participating centers. All patients have given their written informed consent. This trial was registered at ClinicalTrials.gov (NCT01956552). The ESOPE study was approved by the Institut Curie review board and ethics committee (reference number: 2010-A00781-56).

Conventional biopsy procedures, pathological analyses, tissue preparation process, and DNA extractions are detailed in the supplementary methods (Additional file 1). All pathological, immunohistochemical, and genomic analyses were centrally performed at Institut Curie.

### Endpoints

The primary endpoint was to assess the phenotype discordance rate between PT and matched M in patients with first metastatic progression of breast cancer, as defined by centralized pathology analyses. Main secondary endpoints were clinical utility based on the proportion of patients for whom the biopsy led to a change of therapeutic decision for first-line treatment and analysis of genomic landscapes and theranostic input, as a result of the sampling feasibility which remained to be evaluated at the time the study protocol was written (2009). Overall survival was evaluated as a post hoc endpoint.

### Identification of somatic mutation by targeted and whole-exome sequencing

After DNA extraction, 82 paired PT/M FFPE samples were available for sequencing analysis. Targeted sequencing was applied to a custom-made panel of 91 “breast cancer-specific” genes selected for their involvement in breast cancer (Additional file 2: Table S1). This “BreastCurie” panel included the most frequently mutated genes (mutation frequency more than 1%) in breast cancer from TCGA and genes with potential therapeutic targeted mutation (Additional file 1) [16]. Briefly, after preparation of DNA libraries, targeted NGS was performed using Illumina Hiseq2500 technology according to the manufacturer’s instructions (Illumina, San Diego, CA, USA). Sequence data were aligned to the human reference genome (hg19) and we executed variant calling to detect SNV and indels. We retained variants observed at a frequency lower than 0.1% in population (dbSNP or 1000Genome database) and with an allele frequency superior to 5% and covered by 100 reads minimum. Potential pathogenicity of identified variant and druggable genes were selected using multiple public databases. Specifically, ESR1 pathogenic mutations were confirmed by digital droplet PCR (Additional file 1). Next, whole-exome sequencing was performed on 30 pairs of primary tumors and metastases, and their match germline DNAs. Libraries were prepared using SureSelect Human Clinical Research Exome Regions (Agilent) and sequenced on an Illumina HiSeq2000 genome analyzer, generating 2 × 100 bp paired-end reads. For three of the de novo patients (#1, #2, and #11), we downloaded Fastq file from Sequence Read Archive (SRA, SRP055001) [17]. All sequencing analyses flow charts are detailed in the Supplementary Methods.

For each patient with sequencing results, selected variants were assigned to one of the following categories: shared between primary tumor and matched metastasis, private to the primary tumor, or private to the metastasis.

### Mutational signature analysis

The mutational signatures of variants classified as either shared or private were generated separately. Both silent and non-silent SNVs were used as input to Deconstructsigs [18]. This R package compares the profiles of 96 trinucleotides mutations count to the 30 signatures found in the COSMIC classification in order to determine the implication of known mutational processes on each tumor sample [19]. We then merged signatures with the same biological meaning to enhance their weight classification.

### Statistics

Overall, 130 patients were recruited. With 84 PT/M matched pairs, we are able to estimate this proportion with an accuracy of ± 6% (corresponding to a 12% confidence interval centered on the observed rate). Qualitative variables were described with frequency and proportion. Cohen kappa coefficient was used to describe the agreement between local and central laboratory. Confidence interval of the kappa coefficient was obtained using a bootstrap procedure. Median follow-up was estimated using the inverse Kaplan Meier method. Overall survival (OS) was defined as the time elapsed from the inclusion in the study to death. Alive patients were censored to their last known contact date. Survival rates and curves were estimated using Kaplan Meier method and compared with the log-rank test. Univariate Cox proportional hazard models was performed and hazard ratios and their associated 95% confidence intervals were calculated. Variables with a *p* value for the likelihood ratio test no greater than 0.05 in univariate analysis were included in the multivariate model. Backward selection was used to establish the final multivariate models. All analyses were done using data obtained up to November 2018. These analyses were done with R 2.13.2 software.

Regarding molecular analysis, statistics were performed using GraphPad Prism (version 7.01) software. Comparison between PT and M mutations was performed using two-sided Fisher’s exact test paired or unpaired as appropriate. Two-way ANOVA were performed to compare mutational profiles according to several subgroups as tumor molecular subgroup, met sites, met onset time, or phenotypical change. The results were considered statistically significant at a *p* value < 0.05 (*), < 0.01 (**), < 0.001 (***), or 0.0001 (****).

## Results

### ESOPE cohort description and primary outcome

Between November 10, 2010 and September 30, 2013, 130 patients were included, of whom 123 were eligible (Table 1). Data lock was December 31, 2019. Briefly, most patients had an initial high-risk tumor, about 80% had received adjuvant systemic therapy, and 23 (19%) had de novo metastatic disease. Median time to first metastatic event was 42 months (range 0–270). Most patients (61%) had polymetastatic disease. Median time interval between metastasis diagnosis and biopsy was 21 days (0–118). Of the 117 patients who underwent a biopsy, 105 (88%) had samples suitable for IHC analysis (Additional file 3: Fig. S1). Most frequent sites of biopsy were liver (*n* = 53, 45%), bone (*n* = 13, 11%), lung (*n* = 12, 10%), and lymph node (*n* = 11, 9). After central review, there was on both PT and M an excellent agreement between local and central laboratories for ER, PR, and HER2 status, with kappa values varying from 0.78 to 1. Out of 86 evaluable patients, 34 and 54 (39.5% and 62.8%) had detectable CTC, at the 1 cell/7.5 mL and 5 cells/7.5 mL thresholds, respectively. In 78 patients with a completely evaluable subtype, luminal A tumors were the ones to change the most the phenotype, in accordance with previous reports [7, 20]. The primary outcome evaluation at data lock showed that luminal A PT (*n* = 21) developed a more aggressive phenotype on M in 11 patients (52%), of which 7 patients (33%) became luminal B, 2 patients (9.5%) became TN, and 2 patients (9.5%) became HER2. Luminal B PT (*n* = 26) moved to TN tumor in M in 2 patients (8%) and luminal A tumor in 3 patients (11%). Interestingly, luminal A metastases appeared significantly later than luminal B metastases (88 m vs 42 m, *p* = 0.0216), while PT subtype did not significantly influence time to metastasis despite a time trend favoring luminal A tumors (61 m vs 41 m, *p* = 0.2913). None of classical clinical parameters was significantly associated with subtype change.

**Table 1:** Population characteristics at the primary tumor and metastatic settings

			Patients	
			***N***	%
**PT**	Tumor size	pT0, pT1	39	38
		≥ pT2	61	60
		pTx	2	2
		NA	1	1
	No surgery for PT		20	16
	Node invasion*	pN0	38	36
		pN+	65	64
	Histo-prognostic grade*	1	12	10
		2	45	37
		3	63	51
		NA	3	2
	PT treatment	Neo adjuvant chemotherapy	42	34
		Adjuvant chemotherapy	56	46
		Adjuvant endocrine therapy	65	53
		Adjuvant anti HER2 therapy	14	11
**M**	Age	median (min-max), years	57	(28–81)
	Time PT-M	median (min-max), months	42	(0–270)
	M onset	De novo M	23	19
		Late M	100	81
	Number of M sites	1 metastatic site	48	39
		> 1 metastatic site	75	61
	M sites	Liver	74	60
		Bone	53	43
		Lymph node	54	44
		Lung/pleura	42	34
		Skin	9	7
		CNS	6	5
		Ovary	3	2

*PT* primary tumor, *M* metastases, *CNS* central nervous system

**Fig. 1:**
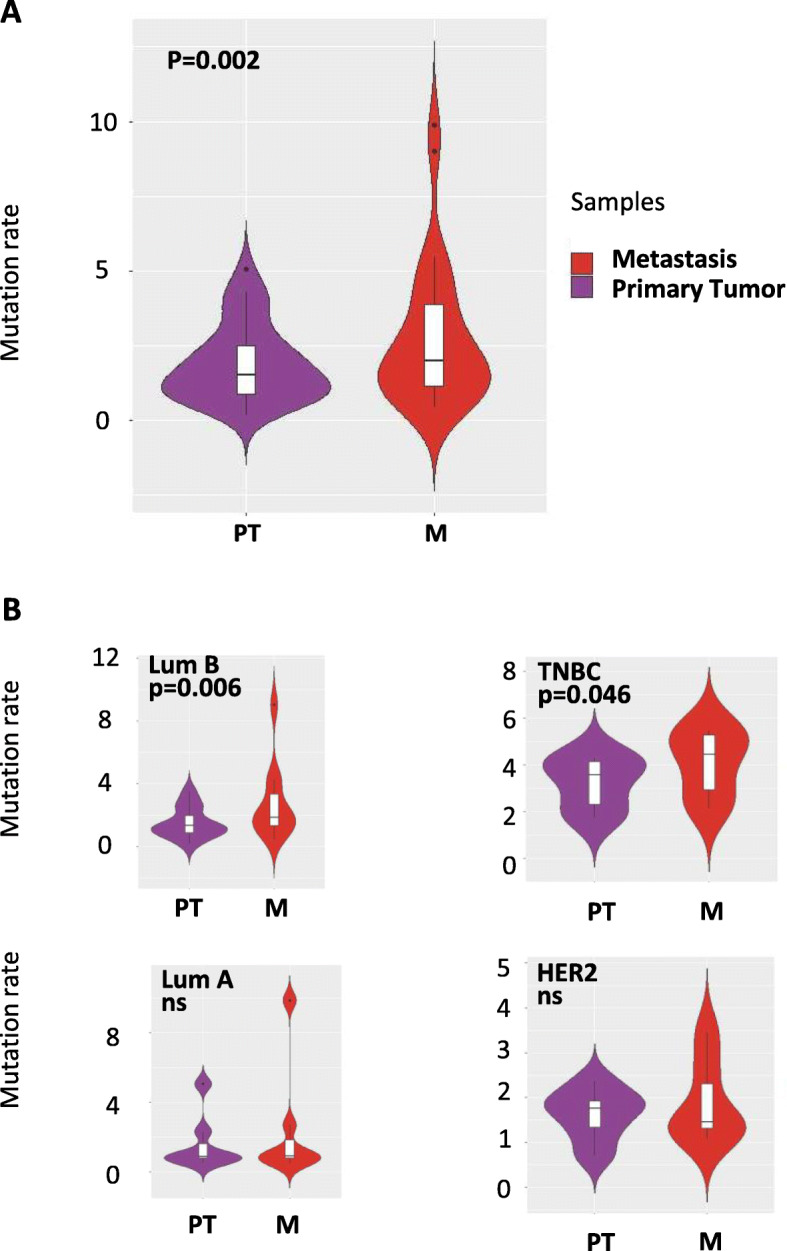
Mutational load in breast cancer primary tumors and their metastatic counterparts by whole-exome sequencing. **a** Average mutation rate in primary tumors (PT) and their matched metastatic tissue (M). **b** Average mutation rate in primary tumors (PT) and their matched metastatic tissue (M), according to the subtype of the primary tumor. The mutational burden in all paired samples (**a**) and by molecular subtypes (**b**) is represented as the number of mutations per megabase of sequence. The violin plots represent the distribution of the data. Asterisks indicate a statistically significant difference among TNBC and luminal B patients (Wilcoxon rank-sum test false discovery rate)

In order to analyze the variations in genomic landscape and their potential theranostic input (secondary outcome, genomic analyses), targeted sequencing was performed on 82 pairs (164 samples) of matched FFPE PT/M samples [21]. Fresh-frozen tumor tissue was available for whole-exome sequencing from 30 individuals, i.e., 60 tumor biopsies corresponding to the primary tumor and a matched metastatic lesion together with a blood sample for germline genetic baseline determination (Additional file 2: Table S2 and additional file 3: Fig. S2).

### Mutational load and mutational landscape of primary tumors and their matched therapy-naïve first metastases

Analyzable matched targeted sequencing results were obtained for 67 patients (82% of 82 pairs) [21]. Sequencing was applied to a custom-made panel of 91 “breast cancer-specific” genes selected for their involvement in breast cancer (Additional file 2: Table S1). The median depth was 892X (minimum 100X in 97% of samples). Overall, 243 mutational events (182 SNVs, 61 indels) were identified in 47 genes. There were 123 unique mutations (91 SNVs, 32 indels). The panel was informative for 62 patients (93%) with at least one mutation in either the tumor or the metastatic sample and a median of 2 mutations per sample (range 0–8). Based on the 47 mutated genes of the analyzed panel, a similar mutational load between PT and M samples was observed with 118 mutations in PT and 131 in M. The median number of mutations was not different between PT and M (Additional file 3: Fig. S3A). We observed 89 shared mutations between PT and M (concordance: 59%), while 41% appeared to be private (65 mutations). The number of shared mutations per sample was significantly higher than the number of private mutations (*p* = 0.01) (Additional file 3: Fig. S3B). Remarkably, a trend to a higher number of private mutations was observed in the metastatic samples (26 private mutations in PT, 39 private mutations in M) (Additional file 3: Fig. S3C).

In 30 patients, we subjected the tumor biopsies and matched constitutional DNA samples to whole-exome sequencing with an average depth of 160× and 70× respectively [22]. Overall, we detected 3453 non-synonymous, somatic coding variants (that is, missense, nonsense, indel, splice) (median 62.5, range 6–347 per biopsy). In accordance with previous reports [10, 13, 23, 24], when analyzing the exome, we observed a significant increase in the number of mutations in metastases relative to primary tumors, suggesting an accumulation of genetic alterations during the metastatic process. The average mutation rate was 1.89 mutations/Mb for primary breast tumors and 2.74 mutations/Mb for metastases (*p* = 0.002, Fig. 1a).

The overall mutation burden of metastatic lesions was significantly higher than primary tumors in luminal B and in TNBC breast cancer patients, whereas there was no difference among luminal A and HER2+ patients (Fig. 1b). Across all paired tumor samples, we detected 1472 events shared between primary tumors and metastases, 587 private to primary tumors, and 1397 private to metastases.

We then analyzed the PT and M molecular profiles, as defined by targeted sequencing, according to their respective IHC-defined tumor subtypes. Patients with sequenced samples had the same clinical characteristics and subtype proportions as the whole population enrolled in the trial. The number of mutations in patients stratified by molecular subtype of the primary tumor is presented in Additional 3 (Fig. S4A), suggesting that by targeted sequencing there is no difference in mutational burden between PT and M in the different subtypes when considered as a whole. However, luminal B, Her2, and TN tumors were significantly enriched in shared mutations while luminal A tumors appeared to be more heterogeneous with as many private as shared mutations in metastases (Additional file 3: Fig. S4B). Overall, mutational burden was similar between tumors and metastases for tumors with and without phenotypic evolution (Additional file 3: Fig. S4C), but private mutations were significantly less frequent in tumors without phenotypic changes whereas a similar load of shared and private mutations was observed in tumors with a change of subtype (Additional file 3: Fig. S4D).

**Fig. 2:**
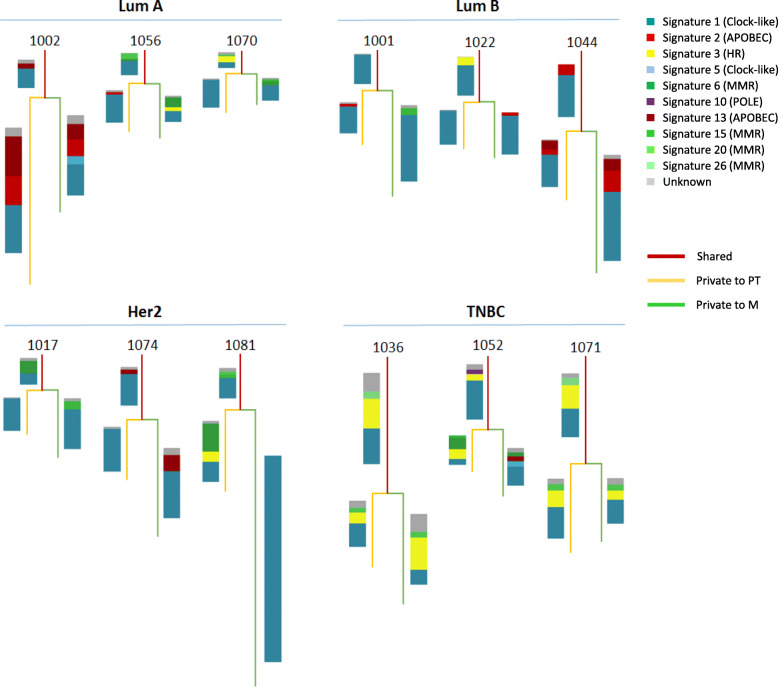
Examples of phylogenetic trees of different tumor subtypes, based on mutational signatures. The mutational signatures were generated separately for variants shared between PT and M or private to each sample. The relative contribution of the signature were then annotated to the phylogenetic tree of 12 breast cancers cases of all 4 subtypes (luminal A, luminal B, Her2, and TNBC). HR, homologous recombination; MMR, mismatch-repair deficiency

We then took advantage of the WES data to test the mutational signatures present in each subtypes of paired tumors and metastases. We found that luminal A tumors give rise to metastases exhibiting a higher number of mutational signatures suggesting the involvement of a wider range of mutational processes in the evolution of these tumor subtype (Additional file 3: Fig. S5). Representative examples of the signatures generated by the shared and private mutations are annotated to the phylogenetic trees of tumors of different subtypes (Fig. 2). Of note, clock-like signature was the major contributor of private mutations in luminal B, Her2, and TN tumors but not in luminal A tumors (> 50% in 74% versus 28%, respectively, *p* = 0.03). In addition, the APOBEC signatures (S2 and S13) seem to emerge late during tumor evolution, regardless of the tumor subtype. Variants contributing to these signatures are predominantly detected as metastatic private variants as compared to shared mutations (43% versus 17%, respectively, *p* = 0.02).

**Fig. 3:**
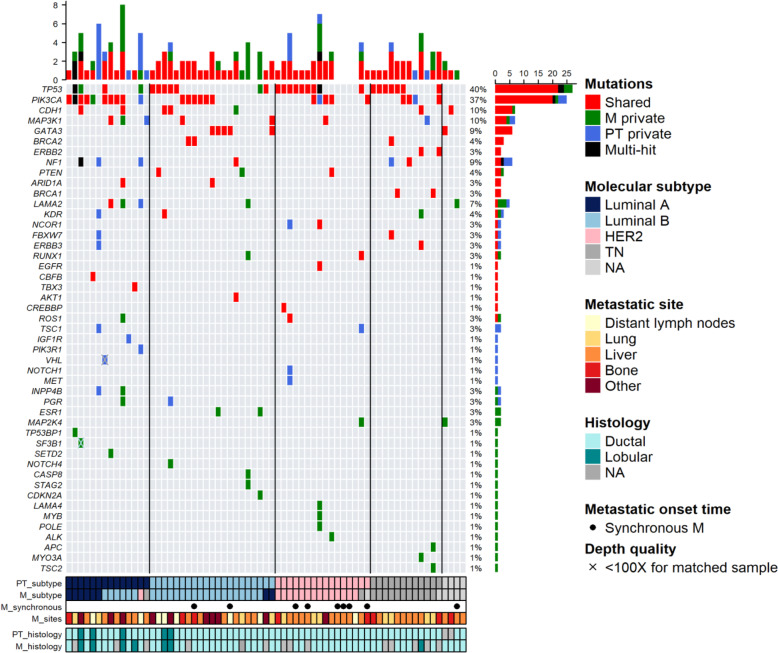
Mutational landscape and mutational load in matched primary tumors and metastases. The respective mutational landscape of primary tumors and matched metastases is shown as captured by targeted sequencing, according to primary tumor subtype and metastatic site. Gray cells indicate a sequencing depth < 100×

Looking at targeted sequencing results, *TP53* and *PIK3CA* were the most frequently mutated genes, with 40% (*n* = 27) and 37% (*n* = 25) of mutated patients respectively (Fig. 3). *CDH1*, *MAP3K1*, *NF1*, *GATA3*, and most interestingly *LAMA2* were mutated in more than 5% of samples. *TP53* and *PIK3CA* were the most frequently shared mutated genes (*n* = 22, 80.5%, and *n* = 20, 80% of mutated patients, respectively). Private mutations in PT in more than two patients were seen in *NF1*, *PIK3CA*, *MAP3K1*, and *TSC1* genes. Private mutations in metastasis in more than two patients were detected in *TP53*, *PIK3CA*, *ESR1*, *MAP2K4*, and *LAMA2* genes. Only 5 patients had no detectable mutations in the metastasis with the present gene panel. Mutational frequencies in primary tumors and metastatic tissues are shown in Additional file 3 (Fig. S6).

Some patients exhibited very peculiar mutational patterns (Fig. 3). For instance, patient #37 (a liver luminal B metastasis at 3 years) exhibited four mutations, all restricted to the metastasis, in *STAG2*, *CASP8*, *RUNX1*, and *LAMA2*. Patient #36 had mutations in seven different genes, in a late lung HER2 overexpressing metastasis. This patient had two very interesting features: the lung metastasis harbored a private mutation in *POLE*, along with mutations in *MYB* and *LAMA4*, but also multiple mutations in *TP53*, which might be related to *POLE* mutations. Patient #35 had an infiltrating lobular carcinoma luminal A PT which moved to a luminal B ovarian metastasis 2 years after diagnosis, with shared mutations in *PIK3CA*, *ARID1A*, and *CDH1*. The ovarian metastasis harbored no less than 8 different mutations, of whom 5 were M restricted, in *MAP3K1*, *ROS1*, *LAMA2*, *INPP4B*, and *PGR*. Only 5 patients in the ESOPE cohort had a lobular carcinoma, precluding further specific analyses.

Our data also suggest that luminal A primary tumors are associated with a higher level of both subtype change and genomic divergence as soon as the first metastatic event than other primary tumor subtypes. Of note, no specific mutation was associated with a given metastatic subtype, except luminal A metastases which had a higher likelihood of *PIK3CA* mutations than other subtypes (62.5% vs 26.9%, *p* = 0.095). *TP53* mutations were more frequently recorded in HR-negative tumors (64% vs 26.8%, *p* = 0.003). Taken together, these data strongly suggest that beyond very commonly mutated genes, each patient has an individual pattern of additional mutations in driver genes in the metastatic tissues.

### Driver genes and theranostics

**Fig. 4:**
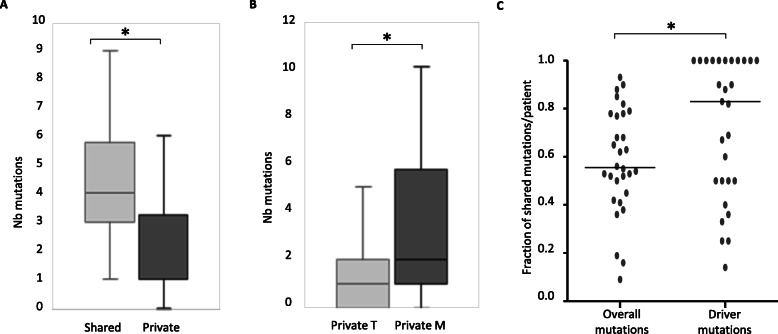
Number and distribution of mutations in driver genes as captured by whole-exome sequencing. **a** Number of shared (light gray) and private (dark gray) mutations in driver genes. *p < 0.01. **b** Number of private mutations in driver genes, in the primary tumor (light gray), or in metastases (dark gray). *p < 0.01. **c** Fraction of overall or driver mutations detected in both primary tumors and metastases. *p<0.01 Lines correspond to the mean

Focusing on driver genes detected by WES [15, 23, 25–29], we determined that mutations affecting cancer drivers tended to be shared by primary tumors and metastases. In agreement with targeted NGS results, we observed that the median number of shared driver mutations per sample was significantly higher than the median of private ones (*p* = 0.03, Fig. 4a). Moreover, private driver mutations are more frequent in metastases than in primary tumors (*p* = 0.02, Fig. 4b). In other words, the proportion of shared driver mutations in ESOPE samples exceeded the proportion of the overall number of shared mutations (median of 83% versus 56%; *P* = 0.001) (Fig. 4c).

Per protocol, the investigators prospectively based their treatment decision for first-line therapy based on local pathology reports (shown as highly concordant with central review results) and treatment decision were available in 77 cases (secondary outcome, clinical utility). They used combined PT and M pathological data for 53 patients with matched PT and M (69%), M data only for 21 patients (27%), and PT data only for 3 patients (4%). IHC subtype discordance between PT and M significantly influenced treating physicians decisions towards decisions based on M characteristics (*p* = 0.0062, Fisher exact test).

Among the 47 mutated genes identified by targeted sequencing, 21 were considered targetable by emerging or available drugs. First, considering shared druggable mutations, 46% (*n* = 31) of patients could be treated for both first disease and relapse. Among those samples, 21 were selected because of *PIK3CA* activating mutation and 10 for other target alterations (Fig. 3). Shared druggable genes were *BRCA2*, *PTEN*, *BRCA1*, *ERBB2*, *NF1*, *KDR*, *AKT1*, *EGFR*, *ERBB3*, *FBXW7*, *CREBBP,* and *ESR1*. Seven patients harbored 2 shared targetable genes, mainly with a *PIK3CA* or *BRCA2* mutation, which imply a decisional algorithm for the choice of treatment. Interestingly, sequencing metastasis revealed for 13% (*n* = 9) of patients at least one additional druggable target in *PIK3CA*, *PTEN*, *KDR*, *ALK*, *CDKN2A*, *NOTCH4*, *POLE*, *SETD2*, *SF3B1,* and *TSC2*. Among 27 genes with M restricted mutations, 11 were druggable (40%). One patient (1/69 = 1.49%) harbored the D538G mutation of the *ESR1* gene in the metastatic sample. This metastasis was luminal B alike the primary tumor and we noted that the patient had received adjuvant AI endocrine therapy during 5 years. Using digital droplet polymerase chain reaction (ddPCR), we also detected the mutation in the matched primary tumor with an allele frequency lower than in the metastatic sample, at 1% and 23.8% respectively. Conversely, allele frequency of the metastatic sample was similar with ddPCR and targeted NGS.

We then analyzed the WES results to identify druggable genes in both primary tumors and metastases (Additional file 3: Fig. S7A). Shared druggable mutations where observed in 63% (42/67) of patients. Metastatic-specific mutations in druggable genes where detected in 33% of the cases (22/67). Overall, and based on the landscape of altered druggable genes either shared or specific to the metastatic tissue, it appears that a wide range of targeted therapies might be used as soon as the first metastatic event in advanced breast cancer (Additional file 3: Fig. S7B).

### Prognostic impact of tumor subtype and mutational landscape

**Fig. 5:**
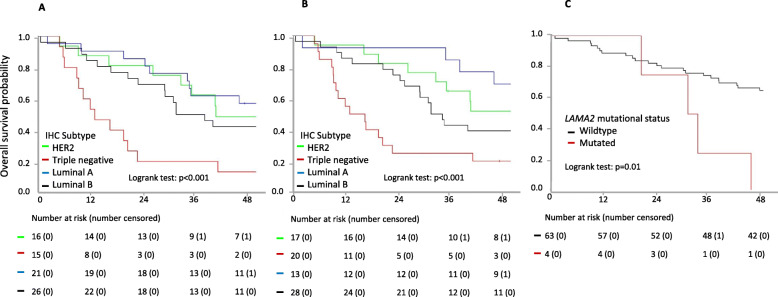
Overall survival. Numbers of patients at risk are indicated beneath the curves. **a** Overall survival according to primary tumor IHC subtype. **b** Overall survival according to metastasis subtype. **c** Overall survival according to LAMA2 mutational status

Overall survival was first analyzed for 78 patients who had centralized PT and M IHC analyses. When considering PT subtype, patients with TN tumors had a worse prognosis compared to other patients who had a very similar overall survival (Fig. 5a, median OS was 13 months 95%CI [9; 41] for TN, 38 months 95%CI [29; not reached (NR)] for luminal B, 56 months 95%CI [35; NR] for luminal A and 41 months 95%CI [33; NR] for HER2; *p* = 0.0009). Patients with luminal A metastases had the best clinical outcome (median OS was 77 months 95%CI [46; NR] for luminal A, and was 86 months 95%CI [35; NR] for HER2, 33 months 95%CI [29; 59] for luminal B and 17 months 95%CI [10; 41] for TN, *p* = 0.0002) (Fig. 5b). By univariate analysis, other prognostic factors associated with overall survival were number of metastatic sites (1–2 vs ≥3) and presence of circulating tumor cells (< 5 vs ≥ 5 cells/7.5 ml). By multivariate analysis, only M IHC subtype, the number of metastatic sites and the presence of CTC ≥ 5 at baseline were independent prognostic factors associated with overall survival (Additional file 2: Table S3). Most importantly, PT IHC subtype was no longer a prognostic factor when adding M IHC subtypes to the multivariate analysis (*p* = 0.8).

The median follow-up for the 67 patients with clinical, pathological, and targeted sequencing information was 64 months (range 35–86). Median OS was 40 months (29–58) and median PFS was 11 months (9–17). Based on univariate analyses (data not shown), two multivariate models for PFS prognostication were built. The first model showed that the M molecular subtype, the number of M sites, and the number of mutations in the M sample had independent PFS prognosis value. Adding pathway mutation information to the model suggested that mutations in the MAPK pathway had a strong poor prognosis value (Additional file 2: Table S4). A similar approach for OS confirmed the strong prognostic impact of key clinical characteristics (sites and number of metastatic sites) and M subtype, and, most interestingly, along with the occurrence of mutations in the *LAMA2* gene which carry a very poor prognosis (Table 2 and Fig. 5c).

**Table 2:** Overall survival multivariate analysis

	Categories	***N***	HR	95%CI	***P*** value
**Visceral metastases**	No	8	1		0.01
	Yes	52	6.57	[1.17; 36.97]	
**Metastases molecular subtype**	Luminal A	8	1		< 0.001
	HER2	15	1.29	[0.32; 5.18]	
	Triple negative	13	10.47	[2.73; 40.17]	
	Luminal B	24	3.49	[1; 12.19]	
**Number of metastatic sites**	1–2	37	1		0.002
	3+	23	2.9	[1.5; 5.62]	
**LAMA2 mutation in metastasis**	Wild type	58	1		0.043
	mutated	2	8.67	[1.43; 52.4]	

### Molecular landscape of de novo versus late metastases

**Fig. 6:**
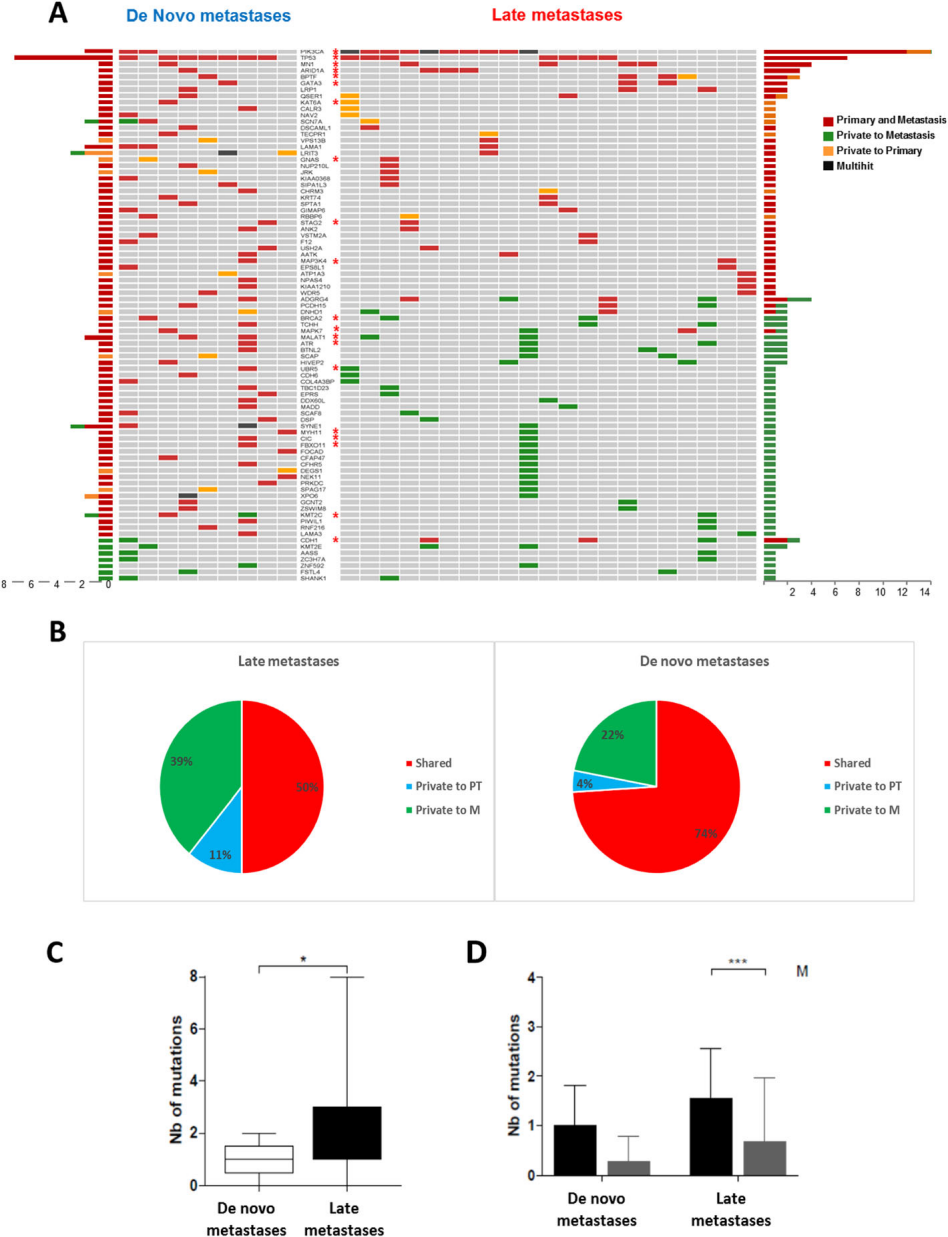
Mutational profile of de novo versus late metastases. **a** Landscape of private versus shared mutations in primary tumors and their matched metastatic tissue, according to metastasis onset pattern. **b** Pie plot showing the proportion of private versus shared mutations in primary tumors and their matched metastatic tissue. **c** Box-plot showing the mutation number in late versus de novo metastases. White box: de novo metastases; black box: late metastases. *p < 0.01. **d** Box-plot showing the median of shared and private driver mutations identified in late and de novo breast cancers. Dark boxes: shared mutations; gray box: private mutations; ***p < 0.0001

We then looked at the relationship between disease-free interval and mutational profile. Metastases were considered de novo when they occurred at the same time as the diagnosis of first disease or less than 6 months later. Completely evaluable subtype was available for 11 and 58 patients of de novo and late metastases respectively. Most interestingly, only one change of subtype between PT and M was observed in the 11 patients with de novo metastases (luminal A, *n* = 1; luminal B, *n* = 2; TNBC, *n* = 2 and HER2 *n* = 6). Figure 6a, b strikingly illustrates how mutations restricted to metastases are mostly observed on late metastases, while most mutations are shared between the primary tumor and de novo metastases. De novo and late metastases had a median number of 1 and 2 mutations, respectively (*p* = 0.04) as observed by targeted sequencing (Fig. 6c). The metastasis-specific mutation number is significantly higher in late than in de novo metastases (Fig. 6d), adding further evidence of the genomic similarity between primary tumors and their synchronous, de novo, metastases.

## Discussion

To the best of our knowledge, ESOPE is the first multicenter prospective study with centralized pathological comparative review and targeted genomic analysis of primary tumor and systematically matched untreated metastatic tissue in a representative population of breast cancer patients at first metastatic event. Overall, we confirm the feasibility in this specific clinical setting of this approach, which has already been shown in more advanced disease [15]. Our study showed discordance in expression between matched PT and M of 17% for ER, 38% for PR, 4% for HER2, and 29% for Ki67 after centralized review. These results are in line with other recent prospective studies [30–32]. Discordance for Ki67 was analyzed in only 4 retrospective studies, with cut-off varying from 14 to 50%, and ranged from 18 to 62% [33–36]. When considering clinical decision-making, 27% of physician decisions were based on M, rather than PT, pathological data. Furthermore, metastatic biopsy allows the diagnosis of non-breast cancers in 4% of cases who had no specific clinical pattern. Other prospective studies showed a change in therapeutic decision in 8 to 21% of cases [7–9, 37]. Interestingly in our study, physician decisions were based on metastatic biopsy when there was PT/M discordance, but also sometimes even in the absence of discordance. For example, confirmation of ER and/or PR status and a low level of Ki67 could allow a clinician to confirm his decision to treat a patient with a first-line endocrine therapy even in presence of a multi-metastatic visceral disease.

NGS and WES analyses revealed targetable shared mutations for 46% and 53% of patients, respectively, where the more frequent target was the PI3K signaling pathway with *PIK3CA*, *PTEN*, and *AKT1* mutations. Considering *ESR1* mutation, the higher level observed in the metastasis of the patient who received previously an endocrine therapy illustrates the concept of selection of the resistant clone in primary tumor [38]. In line with previous studies, we also reported *ESR1* mutations in primary breast cancer at a low allelic frequency [39, 40]. The burden of somatic mutations found in our primary tumors as well as the metastases were consistent with previous reports [10, 11, 15, 41].

Based on an original prospective design of first, untreated, metastatic tissue biopsies, our data bring several novelties pertaining to breast cancer metastatic molecular landscape. In line with previous early findings, in 52% of cases, luminal A tumor in PT changed to a more aggressive subtype in M [9, 30–32]. Moreover, metastases presenting a non-luminal A subtype appeared significantly sooner than luminal A metastases in patients with a luminal A PT, again suggesting heterogeneity of luminal A PT. Understanding the effect of adjuvant therapies on the biology of luminal A tumors (where for example chemotherapy indication remains debatable) could be a key point to determine the optimal medical strategy in the adjuvant setting. Phenotypic instability could also find its origin in genomic characteristics. We thus sequenced 91 genes in 67 matched PT and M samples. Number of mutations did not differ between PT and M samples and most M samples shared a majority of mutations with their matched primary tumors. Our 59% rate of concordant mutations is similar as which observed in 9 exome sequenced pairs of metastatic breast cancer [17], higher than observed by Schrijver et al. [42] with a 341-gene panel in 17 PT-M pairs and lower than reported in Bertucci et al. [10] study about 365 genes sequenced in 23 pairs. Sequencing tools, molecular subtypes, and metastatic sites were all different in these studies. However and most interestingly, the mutational frequencies we report here (Additional file 3: Fig. S6) appear to be different from those observed by Razavi et al. in very advanced, pretreated patients [13]. Indeed, and focusing on the mutational profile in metastases originated from luminal breast cancers, three important trends are unveiled. First, some mutations appear more frequent in late metastases (e.g., *CDH1* 20%; *NF1* 20%, *ESR1* 15%, *ARID1A* 6% in the Razavi series, versus respectively 15%; 9%; 6% and 6% in our data). Second, the mutation rate of core drivers such as *PIK3CA* is very similar (40% in both series). Finally, we observed a decrease in *TP53* mutation rate from 40% (our series) to 20% (Razavi et al.). These data, combined with individual patient-level analysis such as in patient #34, provide some clues as to how the luminal identity and thus cancer fate and patient outcome may be driven by critical genomic alterations. Specifically, inactivating mutations in *ARID1A*, a subunit of the SWI/SNF chromatin-remodeling complex, have been associated with the loss of the luminal phenotype and endocrine resistance [43]. Mutations in *CDH1*, *NF1*, and of course *ESR1* have also been associated with endocrine resistance [10, 13].

We also observed a trend for more frequent mutations and more private mutations in the metastatic tissue. This result is strikingly similar to previous findings in more advanced disease [10, 11, 15], suggesting that key mutational events are acquired very early in the metastatic process. It has indeed been underlined that heterogeneity in mutations in driver genes is minimal between primary tumor and untreated metastases [11]. Global burden of mutations into PT and M samples seemed to be independent of the molecular subtype of primary tumor and metastatic sites in our study. However, luminal A is the only subtype where numbers of private and shared mutations were comparable whereas private mutations were significantly less frequent than shared mutations in all other subtypes. This observation of genomic instability could be linked with the phenotypic instability observed in luminal A subtype. It has also been suggested that ER-positive, HER2-negative tumors displayed an excess of copy number variations [11]. Phenotypically stable tumors do not have less mutation than unstable tumors but they have a higher proportion of mutations that are shared with M, a sign of genomic homogeneity and stability. Interestingly, routine NGS M sequencing reveals 13% of new targetable mutations justifying the biopsy of the metastatic site to optimize clinical management. Among these new targets, we can note the detection of one *POLE* mutation. *POLE* mutations are associated with high tumor mutational burden and are considered as an emerging biomarker for immunotherapy in some tumor models [44]. According to this idea, no less than 8 mutations were counted in the metastatic sample with *POLE* mutation.

Additionally, considering metastatic sites, lung and distant lymph nodes metastatic sites are enriched in private mutations by comparison with liver and bone metastatic sites. We can thus speculate that genomic unstable PT may disseminate more probably in lung and lymph nodes whereas stable PT more probably in liver and bone. However, we did not identify metastasis location-specific alterations in agreement with Schrijver et al. [42]. Finally, the comparison of genomic profiles between late and de novo M revealed a higher number of private mutations in late M. In this situation, it is likely that the clone(s) giving rise to metastases branched early, so tumor and metastasis could cumulate mutations independently over time. Taken together, these observations suggested that genomic instability in primary tumor from patients with late metastases might be more frequent, while the molecular PT/M landscape appears to be strikingly more similar in de novo than in late metastases. This has recently been confirmed by others [11]. The usually better prognosis of de novo metastatic BC might find here a biological explanation [45].

ESOPE is also the first prospective study to our knowledge showing that IHC subtype of breast cancer determined on metastases is an independent prognostic factor of overall survival. Interestingly, overall survival prognostic value of M IHC subtype was stronger than PT subtype. The present results are supported by retrospective studies suggesting that PAM50 defined metastatic subtype is significantly associated with prognosis [46]. Overall, we help define a simple set of powerful prognostic indicators in BC at first metastatic event, combining clinical features and both liquid and solid biomarkers. Independent prognostic value of the baseline CTC count in metastatic breast cancer had been extensively shown in prior studies and is confirmed here [47]. *LAMA2* mutations or expression aberrations, as for other stromal genes, has been repeatedly shown to be associated with a poorer prognosis in breast cancer [48].

### Strengths and limitations

The present study has some limitations. Although prospective and on multicenter basis, accrual was limited. The sample size was adequate for the primary objective, but might be underpowered for secondary and exploratory endpoints. The trial was not randomized, so we cannot definitely prove that biopsy of metastatic disease followed by a tailored therapeutic decision provides an improvement in clinical outcome and only few genes were sequenced. Although the first prospective trials have failed to show the clinical utility of tailored therapy based on metastases genomic characterization [49], new studies are encouraging [50]. Recent studies also spotlighted co-existing targetable genes that could justify combined therapeutic options as triplet therapy [51]. Finally, although this study contributes to the demonstration of temporal heterogeneity in advanced BC and this despite a few set of genes, we have not been able to study spatial heterogeneity of tumor phenotype, because of the refusal of the Ethics Committee to perform several biopsies on the same patient. The study was also not conceived to evaluate temporal variability after onset of M disease. Retrospective analyses suggested a similar discordance rate between successive relapses [52].

## Conclusions

Beyond an excellent concordance between academic centers pathology laboratories, the ESOPE study shows in summary that biopsy of first metastatic event is easily feasible in breast cancer and gives clinically meaningful insights on both prognosis and therapeutic strategy. Thanks to sequencing metastasis, no less than one in every two patients could be candidate for personalized medicine. Prognosis of metastatic breast is confirmed to be driven by a combination of tumor burden and molecular features. We described in depth the mutational landscape of the first metastatic event in breast cancer, further deciphering the difference between de novo and late metastases. Our results strongly support and strengthen current guidelines suggesting that biopsies of metastatic disease in breast cancer patients should be performed as soon as the first metastatic event.

## Supplementary Information

**Additional file 1.** Supplementary Procedures and Methods. This file contains additional methods with regard to tissue handling (sampling, extraction), digital droplet PCR methods, and targeted and whole exome sequencing procedures.

**Additional file 2.** Supplementary Tables. This file contains 4 supplementary tables pertaining to the NGS gene panel, detailed patient characteristics, and additional multivariate analyses.

**Additional file 3.** Supplementary Figures. This file contains 7 supplementary figures further describing the sequencing results.

## Data Availability

Raw genomic data are available at: https://www.ncbi.nlm.nih.gov/sra/PRJNA645212 [22] and at: https://ega-archive.org/studies/EGAS00001004578 [21]. Clinical data are available on reasonable request.
