# Supplementary material for: Molecular features of untreated breast cancer and initial metastatic event inform clinical decision-making and predict outcome: long-term results of ESOPE, a single-arm prospective multicenter study
Source: Genome Med. 2021 Mar 15;13:44. doi: 10.1186/s13073-021-00862-6 (PMC7962302; doi:10.1186/s13073-021-00862-6)
Supplement: Supplementary file 1 — Additional file 1. Supplementary Procedures and Methods. This file contains additional methods with regard to tissue handling (sampling, extraction), digital droplet PCR methods, and targeted and whole exome sequencing procedures. [file 13073_2021_862_MOESM1_ESM.docx]

**Supplementary Methods and Procedures**

*Biopsy procedure, pathological analyses and DNA extraction*

Metastatic samples were obtained by core biopsy or surgical sampling. The most accessible M site was chosen for biopsy after review with a radiologist and a surgeon. Three samples were mandatory for each biopsy: two were formalin fixed and paraffin embedded for local and centralized conventional pathology and IHC analyses, and one was kept frozen for later genomic analyses. Of note, the Ethics Committee did not grant the possibility to perform simultaneous biopsies at different sites in the same patient. Baseline blood samples of each patient were also taken for detection of circulating tumor cells (CTC) with the Cellsearch® method as previously described REF.

After collection, fixation was performed using 4% buffered formaldehyde solution. All biopsies were fixed for 24–48 hours. All PT and M samples were centrally and blindly reviewed at the same laboratory by the same pathologist (BSZ). Quantification of hormone receptors, HER2 and Ki67 was scored using current recommendations REF 10 et (11). Subtypes of breast cancer were defined according to the following classification: luminal (lum) A (HR+, HER2-, Ki67< 20%), lum B (HR+, HER2-, Ki67 ≥20%), HER2 (HER2+, irrespective of HR status), Triple negative (TN) (HR-, HER2-).

Formalin-fixed paraffin-embedded (FFPE) samples obtained before any treatment for advanced BC were available for DNA extraction. Six 10µm-thick sections were subjected to macro dissection by comparison with an H&E stained slide from the same block. DNA was extracted using Nucleospin®8Tissue kit according to the manufacturer’s instructions (Macherey-Nagel). Concentration was assessed by using Qubit dsDNA HS Assay (Life Technologies).

*Digital droplet PCR*

ESR1 pathogenic mutations at codon 538 identified by targeted sequencing were all validated using ddPCR assay. Each ESR1 mutation detected by NGS was verified by ddPCR and ddPCRassay was also applied on each paired sample. Primers and probes were designed and ordered through Integrated DNA Technologies. ddPCR assay was performed using the QX200™ Droplet Digital™ PCR System (Biorad) and allele fraction was calculated using The Bio-Rad QuantaSoft® software (Biorad).

*Targeted sequencing*

A 91 gene-panel relevant to breast cancer (Supplementary table S2) has been selected for multiplex PCR-based amplification targeting all coding exons and intron-exon boundaries from all included genes. 10 ng of genomic tumoral/metastatic DNA were used for PCR amplification using ThermoFischer AmpliSeq Technology followed by subsequent library preparation based on the Truseq nano DNA kit (Illumina, San Diago, CA, USA). Targeted NGS was performed on Illumina Hiseq2500 system using rapid flow cell according to the manufacturer’s instructions (Illumina, San Diago, CA, USA) and paired-end 2x120 nucleotides (PE120) sequencing mode. Expected depth was define around 6 million PE reads per sample to reach an average of 1000X final depth. Sequence data were aligned to the human reference genome (hg19) using Bowtie2 algorithm. SNVs and indels were called using GATK UnifiedGenotyper. We retained variants observed at a frequency lower than 0.1% in population (dbSNP, 1000g) and with an allele frequency superior to 5% and covered by 100 reads minimum. We defined the potential pathogenicity of filtered variants using different public databases (COSMIC [1], Cancer Hotspots [2,3], cbioportal [4,5], tumorportal [6]) and according to the treatment algorithms as previously described [7,8]. Briefly, COSMIC variations that were well established to be activating mutations were considered functionally relevant for oncogenes. Meanwhile, for tumor suppressor genes, nonsense mutations, splice-site mutations, or frameshift insertion/deletions were considered pathogenic. COSMIC missense mutations were considered relevant if they were established inactivating mutations in silico or in the literature. We only retain confirmed pathogenic variants and unknown significant variants for this analysis. Ultimately, selected variants were reviewed using Integrative Genomic viewer [9].

Potential and emerging druggable genes were identified using oncoKB [10], TargetV3 [11], completed by literature [12] and clinical trials extended to other cancers. Selection was done without targeting the exact known hit of genes matching therapies.

*Whole Exome Sequencing (WES)*

1 µg of genomic tumoral/metastatic/germline DNA trios were subjected to DNA shearing using Covaris system and Illumina compatible libraries were performed according to Agilent SureSelect XT2 library protocol. These libraries were then hybridized on dedicated biotinylated RNA probes targeting whole exome sequences (Agilent Clinical Research Exome capture probes). Sequencing of exome selected fragments was performed on an Illumina HiSeq2000 system (high output flow cell) using a paired-end 2x100 nucleotides mode (PE100). Tumoral and germline samples were sequenced to an expected depth of respectively 150M and 50M reads in order to obtain differential depth (>100X versus >30X). For all samples, sequenced reads were aligned to the hg19 human reference genome using Bowtie2 (v2.1.0) (Langmead et al., 2012). Only alignments intersecting the targeted sequence were conserved. Duplicate reads were identified and discarded using Picard Tools (v1.130). Finally, local realignment around small insertions and deletions (indels) and base quality recalibration were performed using the Genome Analysis Toolkit (GATK, v3.5) (McKenna et al., 2010).

Three distinct variants callers were used to identify both the Single Nucleotide Variants (SNVs) and indels, namely UnifiedGenotyper, HaplotypeCaller, and Mutect1 [13,14]. Reference genome positions covered by at least 20 reads in the tumor DNAs and 4 reads in the normal samples were considered for variant calling. All variants were further annotated with ANNOVAR (07/2017 version) [15] and variants with frequencies > 0.1% in 1000 Genomes [16] were filtered. Finally, variants with fequencies less than 3%, or detected in the corresponding germline DNA, were discarded. The resulting high-quality somatic variants were manually inspected using the Integrative Genomics Viewer [17].

Likely driver mutations were identified as those affecting genes of the Cancer Gene Census [18], the Cancer5000 set [6] or from literature review of breast tumors and metastases sequencing studies [6,19,20]. All non-silent mutations were compared with potential driver genes.

To systematically evaluate somatic alterations of potential clinical interest, we used the CancerHotspots and TARGET databases (www.cancerhotspots.org/ and www.broadinstitute.org/cancer/cga/target).

*Mutational signature analysis*

Detected variants were classified as either shared between PT and M or private to each sample, and the mutational signatures of these groups of variants were generated separately. Both silent and non-silent SNVs were used as input to DeconstructSigs [21]. This R package permit to compare the profiles of 96 trinucleotide mutations count to the 30 signatures found in the COSMIC classification in order to determine the implication of known mutational processes on each tumor sample [22].

**References**

1. Tate JG, Bamford S, Jubb HC, Sondka Z, Beare DM, Bindal N, et al. COSMIC: the Catalogue Of Somatic Mutations In Cancer. Nucleic Acids Res. 2019;47:D941–7.

2. Chang MT, Bhattarai TS, Schram AM, Bielski CM, Donoghue MTA, Jonsson P, et al. Accelerating Discovery of Functional Mutant Alleles in Cancer. Cancer Discov. 2018;8:174–83.

3. Chang MT, Asthana S, Gao SP, Lee BH, Chapman JS, Kandoth C, et al. Identifying recurrent mutations in cancer reveals widespread lineage diversity and mutational specificity. Nat Biotechnol. 2016;34:155–63.

4. Gao J, Aksoy BA, Dogrusoz U, Dresdner G, Gross B, Sumer SO, et al. Integrative analysis of complex cancer genomics and clinical profiles using the cBioPortal. Sci Signal. 2013;6:pl1.

5. Cerami E, Gao J, Dogrusoz U, Gross BE, Sumer SO, Aksoy BA, et al. The cBio cancer genomics portal: an open platform for exploring multidimensional cancer genomics data. Cancer Discov. 2012;2:401–4.

6. Lawrence MS, Stojanov P, Mermel CH, Robinson JT, Garraway LA, Golub TR, et al. Discovery and saturation analysis of cancer genes across 21 tumour types. Nature. 2014;505:495–501.

7. Lefebvre C, Bachelot T, Filleron T, Pedrero M, Campone M, Soria J-C, et al. Mutational Profile of Metastatic Breast Cancers: A Retrospective Analysis. Mardis ER, editor. PLOS Med. 2016;13:e1002201.

8. Le Tourneau C, Kamal M, Tsimberidou A-M, Bedard P, Pierron G, Callens C, et al. Treatment Algorithms Based on Tumor Molecular Profiling: The Essence of Precision Medicine Trials. J Natl Cancer Inst. 2016;108.

9. Robinson DR, Wu Y-M, Lonigro RJ, Vats P, Cobain E, Everett J, et al. Integrative clinical genomics of metastatic cancer. Nature. 2017;548:297–303.

10. Chakravarty D, Gao J, Phillips SM, Kundra R, Zhang H, Wang J, et al. OncoKB: A Precision Oncology Knowledge Base. JCO Precis Oncol. 2017;2017.

11. Van Allen EM, Wagle N, Stojanov P, Perrin DL, Cibulskis K, Marlow S, et al. Whole-exome sequencing and clinical interpretation of formalin-fixed, paraffin-embedded tumor samples to guide precision cancer medicine. Nat Med. 2014;20:682–8.

12. Santarpia L, Bottai G, Kelly CM, Győrffy B, Székely B, Pusztai L. Deciphering and Targeting Oncogenic Mutations and Pathways in Breast Cancer. The oncologist. 2016;21:1063–78.

13. Cibulskis K, Lawrence MS, Carter SL, Sivachenko A, Jaffe D, Sougnez C, et al. Sensitive detection of somatic point mutations in impure and heterogeneous cancer samples. Nat Biotechnol. 2013;31:213–9.

14. Poplin R, Ruano-Rubio V, DePristo MA, Fennell TJ, Carneiro MO, Van der Auwera GA, et al. Scaling accurate genetic variant discovery to tens of thousands of samples. bioRxiv [Internet]. Cold Spring Harbor Laboratory; 2018; Available from: https://www.biorxiv.org/content/early/2018/07/24/201178

15. Wang K, Li M, Hakonarson H. ANNOVAR: functional annotation of genetic variants from high-throughput sequencing data. Nucleic Acids Res. 2010;38:e164–e164.

16. Auton A, Abecasis GR, Altshuler DM, Durbin RM, Abecasis GR, Bentley DR, et al. A global reference for human genetic variation. Nature. 2015;526:68–74.

17. Thorvaldsdóttir H, Robinson JT, Mesirov JP. Integrative Genomics Viewer (IGV): high-performance genomics data visualization and exploration. Brief Bioinform. 2013;14:178–92.

18. Futreal PA, Coin L, Marshall M, Down T, Hubbard T, Wooster R, et al. A census of human cancer genes. Nat Rev Cancer. 2004;4:177–83.

19. Razavi P, Chang MT, Xu G, Bandlamudi C, Ross DS, Vasan N, et al. The Genomic Landscape of Endocrine-Resistant Advanced Breast Cancers. Cancer Cell. 2018;34:427-438.e6.

20. Yates LR, Gerstung M, Knappskog S, Desmedt C, Gundem G, Van Loo P, et al. Subclonal diversification of primary breast cancer revealed by multiregion sequencing. Nat Med. 2015;21:751–9.

21. Rosenthal R, McGranahan N, Herrero J, Taylor BS, Swanton C. DeconstructSigs: delineating mutational processes in single tumors distinguishes DNA repair deficiencies and patterns of carcinoma evolution. Genome Biol. 2016;17:31.

22. Alexandrov LB, Nik-Zainal S, Wedge DC, Aparicio S a JR, Behjati S, Biankin AV, et al. Signatures of mutational processes in human cancer. Nature. 2013;500:415–21.
