# Supplementary material for: Molecular features of untreated breast cancer and initial metastatic event inform clinical decision-making and predict outcome: long-term results of ESOPE, a single-arm prospective multicenter study
Source: Genome Med. 2021 Mar 15;13:44. doi: 10.1186/s13073-021-00862-6 (PMC7962302; doi:10.1186/s13073-021-00862-6)
Supplement: Supplementary file 2 — Additional file 2. Supplementary Tables. This file contains 4 supplementary tables pertaining to the NGS gene panel, detailed patient characteristics, and additional multivariate analyses. [file 13073_2021_862_MOESM2_ESM.docx]

**Supplementary Tables**

**Supplementary table S1**: List of the 91 genes relevant to breast cancer that were selected to develop the gene-panel used for targeted sequencing.

**Supplementary table S2**:. Characteristics of patients with targeted sequencing and/or whole exome sequencing results

**Supplementary table S3:** Multivariate analysis of progression-free survival including baseline CTC count.

**Supplementary table S4:** Multivariate analysis of overall survival including pathway alterations.

**Supplementary table S1:** List of the 91 genes relevant to breast cancer that were selected to develop the gene-panel used for targeted sequencing

| Gene name | NM_ |
| --- | --- |
| *CDKN1B* | 004064.4 |
| *PPP2R1A* | 014225.5 |
| *PTPN11* | 002834.3 |
| *RB1* | 000321.2 |
| *TP53* | 000546.4 |
| *STAG2* | 006603.4 |
| *TP53BP1* | 005657.2 |
| *CDK4* | 000075.3 |
| *CDK6* | 001259.6 |
| *TP73* | 005427.3 |
| *CDKN2A* | 000077.4 |
| *APAF1* | 181861.1 |
| *CASP8* | 001228.4 |
| *FBXW7* | 033632.3 |
| *NOTCH1* | 017617.3 |
| *NOTCH2* | 024408.3 |
| *NOTCH4* | 004557.3 |
| *ARID1A* | 006015.4 |
| *CTCF* | 006565.3 |
| *TBL1XR1* | 024665.4 |
| *CREBBP* | 004380.2 |
| *CHD4* | 001273.2 |
| *ESR1* | 000125.3 |
| *FOXA1* | 004496.3 |
| *GATA3* | 002051.2 |
| *NCOR1* | 006311.3 |
| *PGR* | 000926.4 |
| *AR* | 000044.3 |
| *BRAF* | 004333.4 |
| *EGFR* | 005228.3 |
| *FGFR1* | 023110.2 |
| *FGFR2* | 000141.4 |
| *FGFR3* | 000142.4 |
| *FGFR4* | 002011.4 |
| *ERBB2* | 004448.3 |
| *ERBB3* | 001982.3 |
| *MAP3K1* | 005921.1 |
| *ERBB4* | 005235.2 |
| *HRAS* | 005343.2 |
| *IGF1R* | 000875.4 |
| *KRAS* | 004985.4 |
| *MAP2K1* | 002755.3 |
| *MAP2K4* | 003010.3 |
| *MET* | 000245.2 |
| *NF1* | 000267.3 |
| *NRAS* | 002524.4 |
| *PDGFRA* | 006206.4 |
| *RAB40A* | 080879.2 |
| *RET* | 020975.4 |
| *ROS1* | 002944.2 |
| *FLT1* | 002019.4 |
| *KDR* | 002253.2 |
| *ALK* | 004304.4 |
| *DDR2* | 006182.2 |
| *KIT* | 000222.2 |
| *THBS1* | 003246.2 |
| *CDH1* | 004360.3 |
| *LAMA2* | 000426.3 |
| *LAMA4* | 002290.4 |
| *AKT1* | 005163.2 |
| *AKT2* | 001626.5 |
| *AKT3* | 005465.4 |
| *INPP4B* | 003866.2 |
| *MTOR* | 004958.3 |
| *PIK3CA* | 006218.2 |
| *PIK3R1* | 181523.2 |
| *PTEN* | 000314.4 |
| *STK11* | 000455.4 |
| *TSC1* | 000368.4 |
| *TSC2* | 000548.3 |
| *BRCA1* | 007294.3 |
| *BRCA2* | 000059.3 |
| *POLE* | 006231.2 |
| *VHL* | 000551.3 |
| *RUNX1* | 001754.4 |
| *TBX3* | 016569.3 |
| *NFE2L2* | 006164.3 |
| *MYB* | 005375.2 |
| *HIST1H3B* | 003537.3 |
| *SETD2* | 014159.6 |
| *MED1* | 004774.3 |
| *CBFB* | 001755.2 |
| *KEAP1* | 012289.3 |
| *LDLRAP1* | 015627.2 |
| *STMN2* | 007029.3 |
| *MYO3A* | 017433.4 |
| *AGTR2* | 000686.4 |
| *CTNNB1* | 001904.3 |
| *APC* | 000038.5 |
| *SF3B1* | 012433.2 |
| *MYH9* | 002473.4 |

| **Supplementary table S2**: Characteristics of patients with targeted sequencing and/or whole exome sequencing results. | | | | | | | | | | | |
| --- | --- | --- | --- | --- | --- | --- | --- | --- | --- | --- | --- |
|  |  |  |  |  |  |  |  |  |  |  |  |
| **Patient** | | **Primary Tumor** | | | **Metastasis** | | | | **Treatment** | | **Sequencing method** |
| **Case ID** | **Age at diagnosis** | **Stage** | **Tumor subtype** | **Grade** | **Metastatic biopsy** | **Metastasis subtype** | **Time to metastasis (years)** | **Nb metastatic sites** | **Adjuvant therapy** | **Overall survival (years)** |  |
| 1 | 47 | IV | Her2+ | 2 | Liver | Her2+ | 0 | 1 | NA | 6.6+ | Targeted, WES |
| 2 | 61 | IV | Her2+ | 3 | Liver | Her2+ | 0 | 2 | NA | 3.4 | Targeted, WES |
| 3 | 51 | IV | Luminal B | 3 | Bone | Luminal B | 0 | 1 | NA | 6.1+ | Targeted, WES |
| 4 | 50 | IV | TNBC | 2 | Skin | TNBC | 0 | 2 | NA | 5.9 | WES |
| 5 | 47 | IV | Luminal A | 2 | Liver | Luminal B | 0 | 1 | NA | 4.9 | WES |
| 6 | 47 | IV | Her2+ | 3 | Liver | Her2+ | 0 | 1 | NA | 2.7 | Targeted, WES |
| 7 | 70 | II | Luminal B | 2 | Lymph node | Luminal B | 0 | 1 | NA | 3+ | Targeted |
| 8 | 27 | IV | Her2+ | 3 | Bone | Her2+ | 0 | 1 | NA | 3.0+ | Targeted, WES |
| 9 | 46 | IV | Her2+ | 3 | Liver | Her2+ | 0 | 1 | NA | 4.5+ | Targeted, WES |
| 10 | 46 |  | NA |  | Liver | NA | 0 | 1 | NA | 1.5 | Targeted |
| 11 | 53 | IV | TNBC | 3 | Lymph node | TNBC | 0 | 5 | NA | 0.8 | WES |
| 12 | 64 | II | Her2+ | 3 | Liver | Her2+ | 0.4 | 1 | NA | 4+ | Targeted |
| 13 | 50 | III | Luminal B | 3 | Brain | Luminal B | 0.8 | 1 | CT ET RT | 0.6 | Targeted |
| 14 | 67 | III | Luminal B | 3 | Bone | Luminal B | 0.9 | 1 | CT ET RT | 6.3+ | Targeted, WES |
| 15 | 50 | II | TNBC | 3 | Liver | TNBC | 0.9 | 1 | CT RT | 1.1+ | Targeted |
| 16 | 34 | III | Luminal B | 2 | Skin | Luminal B | 1 | 4 | CT ET RT | 0.04 | Targeted |
| 17 | 63 | II | TNBC | 3 | Liver | TNBC | 1 | 1 | CT RT | 1.9 | Targeted, WES |
| 18 | 43 | I | Luminal A | 1 | Liver | Luminal B | 1 | 1 | ET | 3.6+ | Targeted |
| 19 | 59 | II | Her2+ | 2 | Liver | TNBC | 1.2 | 2 | CT TL RT | 0.8 | Targeted |
| 20 | 60 | III | Luminal B | 3 | Liver | Luminal B | 1.2 | 1 | CT ET RT | 1.7 | Targeted, WES |
| 21 | 27 | II | Her2+ | 3 | Liver | Her2+ | 1.2 | 1 | CT TL ET RT | 2.5+ | Targeted |
| 22 | 34 | III | TNBC | 3 | Lung | TNBC | 1.2 | 4 | CT RT | 0.4 | Targeted |
| 23 | 58 | I | TNBC | 2 | Bone | TNBC | 1.5 | 2 | CT ET RT | 1.4 | Targeted |
| 24 | 59 | III | TNBC | 3 | Lung | TNBC | 1.5 | 2 | CT RT | 3.4 | Targeted, WES |
| 25 | 40 | III | Luminal B | 2 | Sus clavicular node | Luminal B | 1.8 | 2 | CT ET RT | 2.7+ | Targeted |
| 26 | 61 | I | Her2+ | 2 | Lung | Her2+ | 2 | 3 | CT TL RT | 1.4 | Targeted |
| 27 | 48 | II | Her2+ | 3 | Brain | Her2+ | 2.1 | 1 | CT TL ET RT | 0.7 | Targeted |
| 28 | 53 | II | TNBC | 3 | Lung | TNBC | 2.2 | 2 | CT RT | 0.5 | Targeted |
| 29 | 34 | I | Luminal A | 2 | Lung | Luminal A | 2.2 | 1 | CT ET RT | 2.9+ | Targeted |
| 30 | 71 | III | TNBC | 2 | Lung | TNBC | 2.4 | 3 | CT RT | 1 | Targeted |
| 31 | 41 | II | Luminal B | 3 | Liver | Luminal B | 2.5 | 1 | CT ET RT | 2.4 | Targeted |
| 32 | 57 | I | Luminal B | 2 | Lymph node | Luminal B | 2.5 | 1 |  | 3.4+ | Targeted |
| 33 | 45 | II | TNBC | 3 | Liver | TNBC | 2.7 | 3 | CT RT | 0.5 | Targeted, WES |
| 34 | 60 | III | Luminal A | 3 | Ovary | Luminal A | 2.7 | 3 | CT ET RT | 1.5 | Targeted, WES |
| 35 | 55 | II | Her2+ | 2 | Lung | Her2+ | 2.7 | 2 | CT TL RT | 2.7+ | Targeted |
| 36 | 54 | II | Luminal B | 2 | Liver | Luminal B | 2.8 | 1 | CT ET RT | 0.9 | Targeted, WES |
| 37 | 46 | II | TNBC | 3 | Liver | TNBC | 2.8 | 1 | CT RT | 1.8 | Targeted |
| 38 | 39 | II | Luminal B | 2 | Lymph node | Luminal B | 3 | 1 | CT ET RT | 1.1 | Targeted |
| 39 | 35 | I | TNBC | 2 | Liver | TNBC | 3.1 | 3 | CT RT | 0.9 | Targeted |
| 40 | 40 | II | Luminal B | 3 | Lung | Luminal A | 3.4 | 1 | CT ET RT | 2.8+ | Targeted |
| 41 | 69 | III | Luminal B | 3 | Muscle | Luminal B | 3.5 | 3 | CT ET RT | 1.9 | Targeted, WES |
| 42 | 53 | III | Luminal A | 2 | Liver | Luminal B | 3.6 | 2 | CT ET RT | 2.9 | WES |
| 43 | 37 | II | Luminal A | 2 | Liver | Luminal B | 3.6 | 3 | CT ET RT | 3+ | Targeted |
| 44 | 35 | II | Luminal A | 1 | Lung | Luminal A | 3.9 | 3 | CT ET RT | 2.2+ | Targeted |
| 45 | 61 | III | Luminal B | 3 | Lymph node | Luminal B | 4 | 2 | CT ET RT | 0.8 | Targeted, WES |
| 46 | 54 | I | Luminal B | 2 | Liver | Luminal B | 4.2 | 2 | CT ET RT | 5 | Targeted |
| 47 | 56 | III | Luminal B | 3 | Liver | Luminal B | 4.2 | 2 | CT ET RT | 3.3 | Targeted, WES |
| 48 | 55 | II | Luminal B | 3 | Liver | Luminal B | 4.2 | 2 | CT ET RT | 2.6 | Targeted, WES |
| 49 | 36 | II | Her2+ | 3 | Bone | Her2+ | 4.3 | 2 | CT TL RT | 2.9 | Targeted |
| 50 | 39 | I | NA |  | Liver | NA | 4.4 | 2 | CT ET RT | 3.3+ | Targeted |
| 51 | 64 | I | TNBC | 2 | Bone | NA | 4.5 | 3 | RT* | 1.6 | Targeted, WES |
| 52 | 52 | II | Her2+ | 2 | Lymph node | Her2+ | 4.5 | 2 | CT TL RT | 4.4+ | Targeted, WES |
| 53 | 72 | II | Luminal A | 3 | Liver | TNBC | 4.6 | 2 | ET RT | 1.4+ | Targeted |
| 54 | 36 | II | Luminal B | 2 | Chest wall | Luminal B | 4.8 | 3 | CT ET RT | 2.6+ | Targeted |
| 55 | 40 | III | Her2+ | 2 | Liver | Her2+ | 4.9 | 2 | CT TL ET RT | 3.4 | Targeted |
| 56 | 57 | II | Luminal B | 3 | Liver | Luminal B | 5.1 | 1 | CT ET RT | 4.4 | Targeted, WES |
| 57 | 45 | I | Luminal B | 3 | Liver | Luminal B | 5.5 | 2 | CT ET RT | 2.4 | WES |
| 58 | 51 | II | Luminal A | 1 | Lymph node | Luminal A | 5.5 | 2 | ET RT | 0.2 | Targeted |
| 59 | 36 | II | Luminal A | 2 | Liver | Luminal B | 5.8 | 1 | CT ET RT | 2.2+ | Targeted |
| 60 | 48 | II | Luminal B | 2 | Lung | Luminal B | 6 | 2 | ET RT | 4.3 | Targeted |
| 61 | 40 | II | Her2+ | 2 | Liver | Her2+ | 6.4 | 1 | CT TL ET RT | 2.7+ | Targeted |
| 62 | 65 | IIII | TNBC | 1 | Liver | TNBC | 6.7 | 2 | CT ET RT | 1.8+ | Targeted |
| 63 | 61 | II | Luminal A | 2 | Ovary | Luminal A | 7.4 | 2 | ET RT | 4.6+ | Targeted, WES |
| 64 | 41 | III | Luminal A | 2 | Liver | Luminal A | 7.5 | 4 | CT ET RT | 4.8+ | WES |
| 65 | 66 | I | Luminal A | 2 | Bone | Luminal A | 8 | 1 | ET RT | 6.3+ | Targeted, WES |
| 66 | 57 | I | Luminal A | 1 | Liver | Luminal A | 8.4 | 2 | ET RT | 3.8 | Targeted, WES |
| 67 | 48 | III | Luminal B | 2 | Muscle | Luminal B | 9.7 | 2 | CT ET RT | 5.2+ | Targeted, WES |
| 68 | 67 | I | NA |  | Bone | NA | 10.2 | 1 | ET RT | 4.6+ | Targeted |
| 69 | 50 | III | Luminal A | 2 | Skin | Luminal B | 11.4 | 2 | CT ET RT | 2.1 | Targeted |
| 70 | 39 | II | Her2+ | 1 | Lung | Her2+ | 12.7 | 1 | CT RT | 2.5+ | Targeted |
| 71 | 38 | I | Luminal A | 1 | Liver | Luminal B | 13.5 | 2 | CT ET RT | 2.1 | Targeted |
| 72 | 46 | II | Luminal A | 2 | Lymph node | Her2+ | 15.1 | 1 | RT | 2.4+ | Targeted |
| 73 | 45 | III | Luminal B | 2 | Chest wall | Luminal A | 17.6 | 1 | RT | 3+ | Targeted |

**Pathological Abbreviations**

IDC: Invasive Ductal Carcinoma

ILC: Invasive Lobular Carcinoma

HER2: Human Epidermal growth factor Receptor 2

TNBC: Triple Negative Breast Cancer

**Treatment Abbreviations**

NA: Not applicable

CT: Chemotherapy

ET: Endocrine treatment

RT: Radiation therapy

TL: Trastuzumab, Lapatinib

*patient denied adjuvant CT

**Supplementary table S3:** Multivariate analysis of progression-free survival including baseline CTC count.

|  | | **RR** | **95%CI** | **p** |
| --- | --- | --- | --- | --- |
| **CTC value** | <5 | 1 |  | <0.0001 |
|  | ≥5 | 5.25 | [2.21 ; 12.50] |  |
| **Number of M sites** | 0-2 | 1 |  | 0.0078 |
|  | >3 | 2.96 | [1.34 ; 6.52] |  |
| **M IHC subtype** | Luminal A | 1 |  | <0.0001 |
|  | Luminal B | 1.38 | [0.30 ; 6.33] |  |
|  | TN | 11.6 | [2.28 ; 58.99] |  |
|  | HER2 | 0.55 | [0.09 ; 3.29] |  |

CTC : circulating tumor cells

M: metastatic

IHC : immunohistochemistry

**Supplementary table S4:** Multivariate analysis of overall survival including pathway alterations.

|  | **categories** | **N** | **HR** | **IC** | **P value** |
| --- | --- | --- | --- | --- | --- |
| **M subtype** | Luminal A | 8 | 1 |  | <0.001 |
|  | HER2 | 15 | 1.47 | [0.56 ; 3.87] |  |
|  | Triple Negative | 13 | 5.97 | [2.2 ; 16.15] |  |
|  | Luminal B | 24 | 3.33 | [1.35 ; 8.2] |  |
| **Number of M sites** | 1-2 | 37 | 1 |  | 0.015 |
|  | 3+ | 23 | 2.09 | [1.17 ; 3.75] |  |
| **MAPKs (in M tissue)** | MAPK wt | 47 | 1 |  | 0.009 |
|  | MAPK mut. | 13 | 2.66 | [1.33 ; 5.34] |  |

M: metastatic

MAPK: mitogen-activated protein kinases

Wt: wild type

Mut.: mutation in the MAPK pathway
