# Supplementary material for: Molecular features of untreated breast cancer and initial metastatic event inform clinical decision-making and predict outcome: long-term results of ESOPE, a single-arm prospective multicenter study
Source: Genome Med. 2021 Mar 15;13:44. doi: 10.1186/s13073-021-00862-6 (PMC7962302; doi:10.1186/s13073-021-00862-6)
Supplement: Supplementary file 3 — Additional file 3. Supplementary Figures. This file contains 7 supplementary figures further describing the sequencing results. [file 13073_2021_862_MOESM3_ESM.docx]

**Supplementary Figures**

**Supplementary Figure S1.** Consort diagram

**Supplementary Figure S2**. Distribution of biopsies in metastatic sites.

**Supplementary Figure S3**. Mutational load in breast cancer primary tumors and their metastatic counterparts by targeted sequencing

**Supplementary Figure S4**. Mutational load in breast cancer primary tumors and their metastatic counterparts according to the primary tumor subtype.

**Supplementary Figure S5**. Mutational frequencies in primary tumors and their metastatic matched tissue.

**Supplementary Figure S6.** Median number of mutational signatures contributing to at least 10% of the molecular profiles of the metastases according to the primary tumor subtype

**Supplementary Figure S7**. Druggable mutations.

**Supplementary Figure S1.** Consort diagram


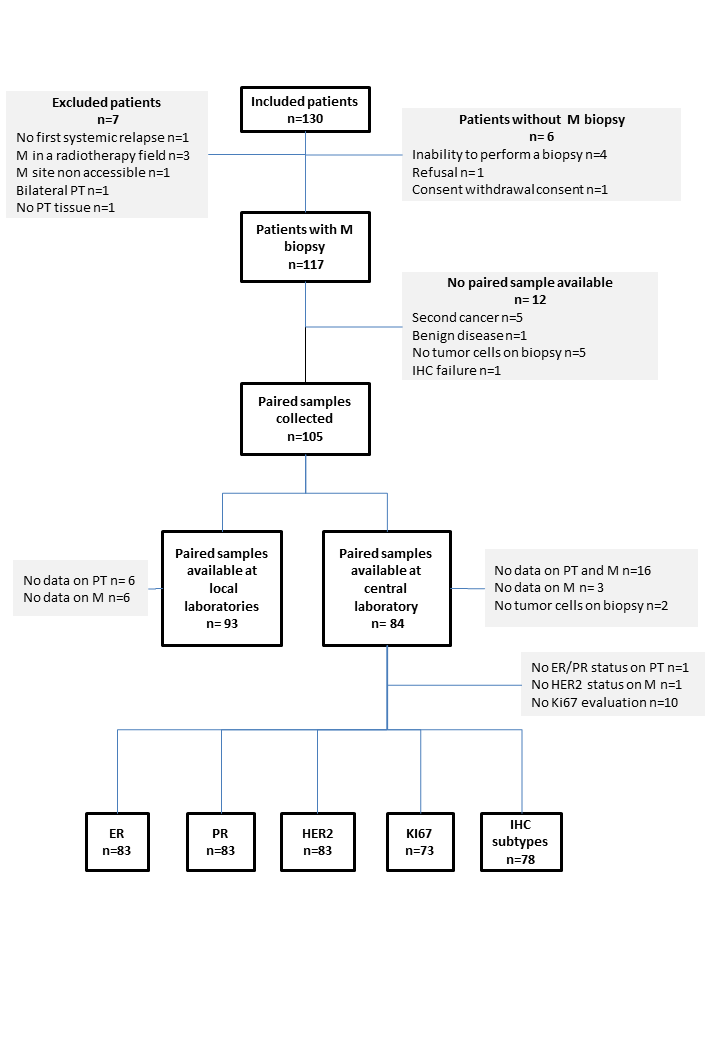


Of 130 included patients, 117 had a biopsy of one metastatic site. Paired samples were analysed locally and centrally. The bottom line of the diagram indicates the final numbers of patients with available matched PT/M central review. IHC subtyping was feasible in 78 patients with central analysis of ER, PR, HER2 and Ki67 expression.

IHC: Immunochemistry - PT: Primary tumor - M: Metastase

**Supplementary Figure S2**. Distribution of biopsies in metastatic sites.

**
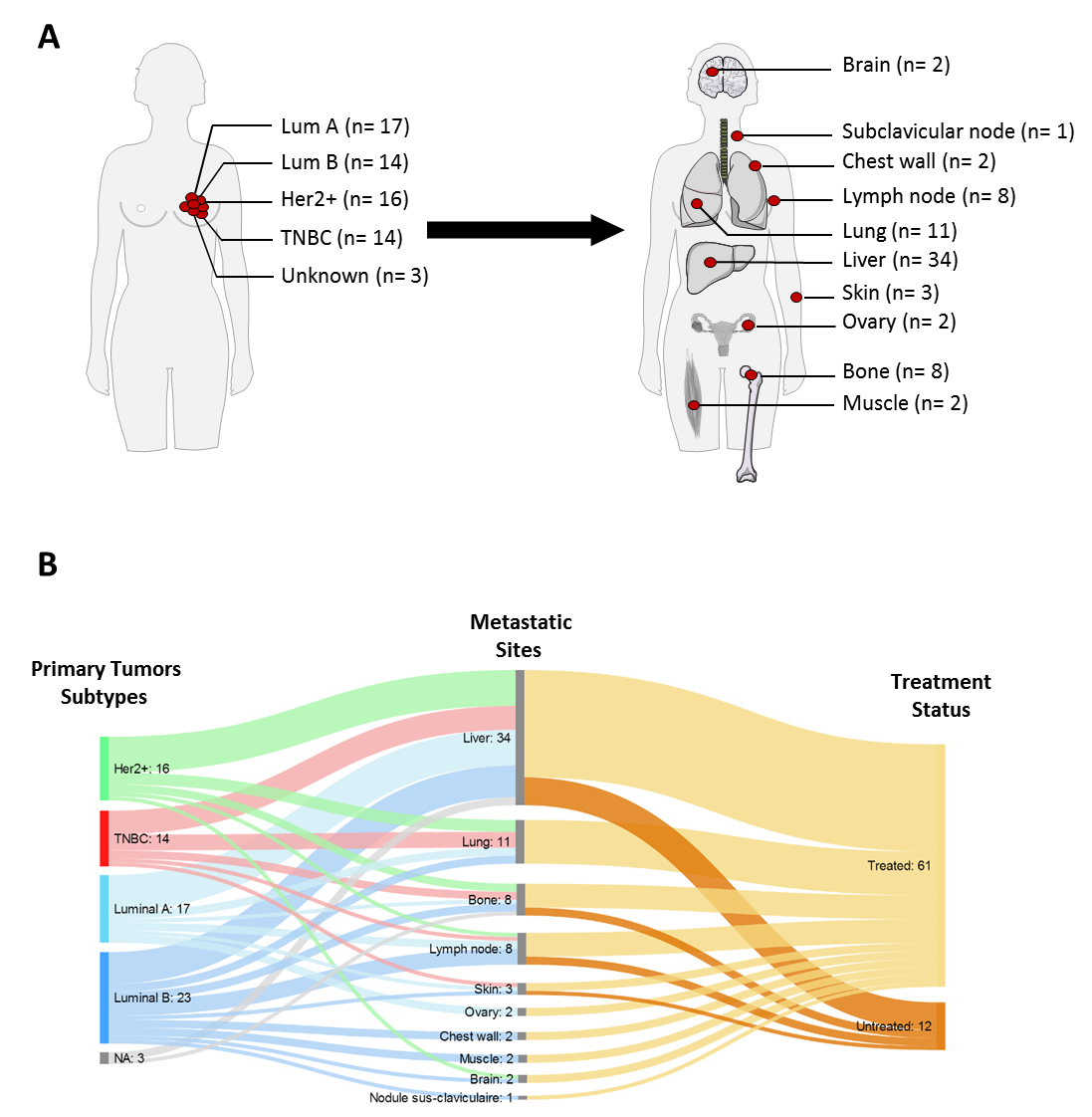
**A. Breast cancer subtypes in ESOPE cohort and corresponding metastatic sites. Number of cases of each types are indicated. B. Sankey diagram of ESOPE patients. 73 primary tumors and paired metastases are depicted. Treatment status for all patients are indicated

**Supplementary Figure S3**. Mutational load in breast cancer primary tumors and their metastatic counterparts by targeted sequencing.

1. Number of mutations per sample in primary tumor (PT) and matched metastatic tissue (M). No difference was recorded.
2. Overall number of of shared (in PT and M) and private (present in either PT or M) mutations. The number of shared mutations per sample was significantly higher than the number of private mutations (*p=0.01).
3. Number of private mutations in either PT or M.

**Supplementary Figure S4**. Mutational load in breast cancer primary tumors and their metastatic counterparts according to the primary tumor subtype, as captured by targeted sequencing.

1. Number of mutations in primary tumors (PT) and their matched metastatic tissue (M), arranged by subtype of the primary tumor.
2. Number of shared mutations in PT and M, according to the subtype of the primary tumor. *p<0.01; **p<0.001; ****p<0.00001.
3. Number of mutations in primary tumors (PT) and their matched metastatic tissue (M), according to subtype change.
4. Number of shared mutations in PT and M according to subtype change. ***p<0.0001; ****p<0.00001.

**A B**

**C D**

**Supplementary Figure S5.** Median number of mutational signatures contributing to at least 10% of the molecular profiles of the metastases according to the primary tumor subtype.

**
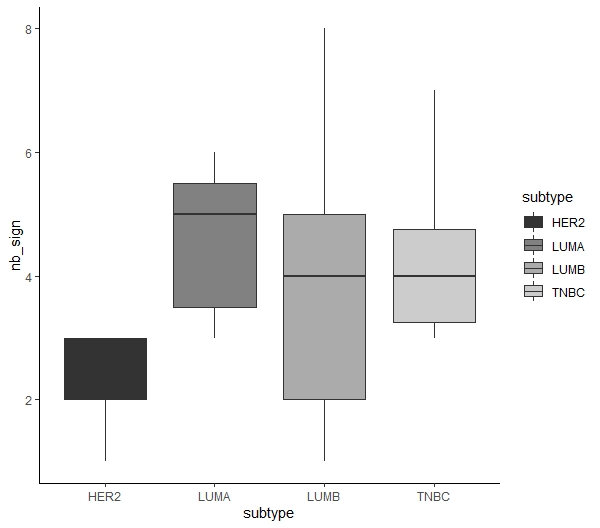
**

**Supplementary Figure S6**. Mutational frequencies in primary tumors and their metastatic matched tissue.

**Supplementary Figure S7**. Druggable mutations.

1. Mutations in druggable genes are either shared between the primary tumor and its matched metastatic tissue (shown in red), or observed only in the metastatic tissue (shown in green).
2. Range of potential druggable pathways that might be harnessed for first line therapy for metastatic breast cancer, as based on molecular profiling of the first metastatic event, and shared by primary tumors and metastases or emerging as private events in the metastatic samples.


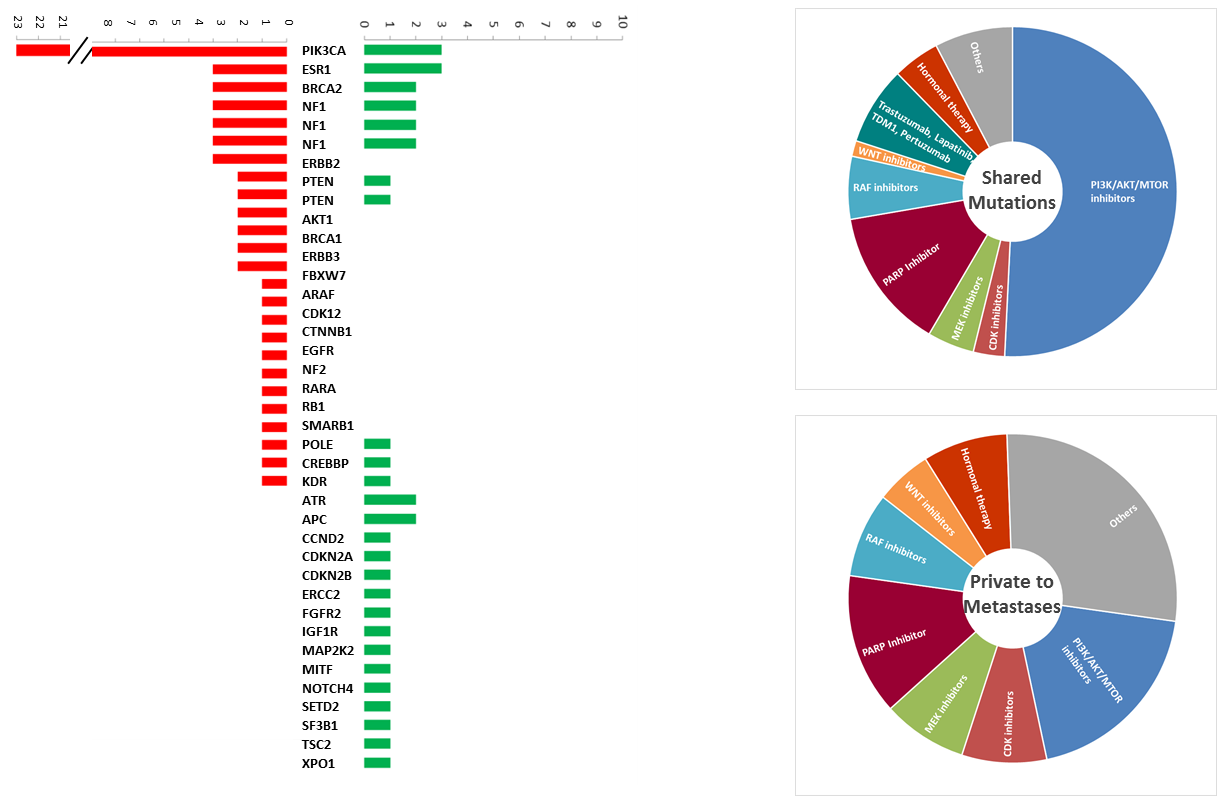
**A B**
